# Deciphering Common Genetic Pathways to Antibiotic Resistance in *Escherichia coli* Using a MEGA-Plate Evolution System

**DOI:** 10.3390/antibiotics14080841

**Published:** 2025-08-20

**Authors:** Nami Morales-Durán, Angel León-Buitimea, Roberto Álvarez Martínez, José Rubén Morones-Ramírez

**Affiliations:** 1 Facultad de Ciencias Químicas, Universidad Autónoma de Nuevo León (UANL), San Nicolás de los Garza 66455, Mexico; nami.moralesd@uanl.edu.mx (N.M.-D.); angel.deb@uanl.edu.mx (A.L.-B.); 2 Centro de Investigación en Biotecnología y Nanotecnología, Facultad de Ciencias Químicas, Universidad Autónoma de Nuevo León, Parque de Investigación e Innovación Tecnológica, Apodaca 66628, Mexico; 3 Laboratorio de Biología Cuantitativa y Sistemas Complejos, Unidad de Microbiología Básica y Aplicada, Facultad de Ciencias Naturales, Universidad Autónoma de Querétaro, Santiago de Querétaro 76123, Mexico; roberto.alvarez@uaq.edu.mx

**Keywords:** antimicrobial resistance (AMR), MEGA-plate system, *Escherichia coli* evolution, antibiotic resistance genes (ARGs), One Health approach

## Abstract

**Background.** Antimicrobial resistance (AMR) poses a significant global health threat, necessitating a deeper understanding of bacterial adaptation mechanisms. **Introduction.** This study investigates the genotypic and phenotypic evolutionary trajectories of *Escherichia coli* under meropenem and gentamicin selection, and it benchmarks these findings against florfenicol-evolved strains. **Methodology.** Utilizing a downsized, three-layer acrylic modified “Microbial Evolution and Growth Arena (MEGA-plate) system”—scaled to 40 × 50 cm for sterile handling and uniform 37 °C incubation—we tracked adaptation over 9–13 days, enabling real-time visualization of movement across antibiotic gradients. **Results.** Meropenem exposure elicited pronounced genetic heterogeneity and morphological remodeling (filamentous and circular forms), characteristic of SOS-mediated division arrest and DNA-damage response. In contrast, gentamicin exposure produced a uniform resistance gene profile and minimal shape changes, suggesting reliance on conserved defenses without major morphological adaptation. Comprehensive genomic analysis revealed a core resistome of 22 chromosomal loci shared across all three antibiotics, highlighting potential cross-resistance and the central roles of *baeR*, *gadX*, and *marA* in coordinating adaptive responses. Gene ontology enrichment underscored the positive regulation of gene expression and intracellular signaling as key themes in resistance evolution. **Discussion.** Our findings illustrate the multifaceted strategies *E. coli* employs—combining metabolic flexibility with sophisticated regulatory networks—to withstand diverse antibiotic pressures. This study underscores the utility of the MEGA-plate system in dissecting spatiotemporal AMR dynamics in a controlled yet ecologically relevant context. **Conclusions.** The divergent responses to meropenem and gentamicin highlight the complexity of resistance development and reinforce the need for integrated, One Health strategies. Targeting shared regulatory hubs may open new avenues for antimicrobial intervention and help preserve the efficacy of existing drugs.

## 1. Introduction

Antimicrobial resistance (AMR) has emerged as one of the foremost global health challenges, projected to cause up to 10 million deaths per year by 2050—surpassing cancer mortality—and impose an economic burden to the global economy exceeding USD 100 trillion [1]. At its core, resistance reflects Darwinian selection: spontaneous genetic variants (whether chromosomal mutations or mobile element acquisitions) that survive antibiotic exposure expand and dominate the population [2]. Excessive and often inappropriate antibiotic use in human medicine, agriculture, and animal husbandry has accelerated this process [3]. Overprescription, patient misuse, and the use of antibiotics as growth promoters in livestock create selective pressures that foster the emergence and spread of resistant strains [4].

A key factor in the evolution of resistance is the phenotypic and genotypic adaptability of bacteria, propelled by high reproduction rates, horizontal gene transfer, and mutations [5]. Bacteria can quickly acquire and disseminate resistance determinants across various lineages and environments [6]. Sub-inhibitory antibiotic concentrations—common in clinical settings with suboptimal dosing and in the environment via runoff—select for resistant phenotypes and enrich multidrug-resistant (MDR) populations [7,8].

Understanding the evolutionary mechanisms behind AMR hinges on elucidating the role of chromosomal loci encoding resistance-associated functions—hereafter referred to as antibiotic resistance genes (ARGs)—which are central to this arms race. Many of these loci are ancient and embedded in basic bacterial functions, but under selective pressure can be co-opted to confer resistance [9]. Efflux pumps, for instance, are highly conserved transport proteins that expel toxic substances, including antibiotics, from the cell [10]. Upregulation or mutations in these systems can amplify resistance [11]. Thus, examining ARGs in both normal physiology and the evolution of resistance is crucial for unraveling AMR and MDR.

Resistance becomes more complex when different antibiotic classes are involved. Carbapenems, including imipenem and meropenem, are last-resort agents against severe Gram-positive and Gram-negative infections due to their broad spectrum, resilience to most β-lactamases, and low cytotoxicity [12]. However, carbapenem-resistant Enterobacteriaceae (CRE) have emerged globally, often linked to high morbidity and mortality [13]. The principal resistance mechanisms involve carbapenemase production, efflux pumps, and porin loss, which prevents antibiotic entry [14,15].

Meropenem is effective against Enterobacteriaceae such as *Citrobacter freundii*, *Enterobacter aerogenes*, *Enterobacter cloacae*, *Escherichia coli*, *Klebsiella pneumoniae*, *Klebsiella oxytoca*, *Morganella morganii*, *Proteus vulgaris*, and *Serratia marcescens* [16]. By binding to PBPs and blocking cell wall synthesis, it causes bacterial cell death [17]. The emergence of meropenem-resistant strains presents a major threat, restricting treatment to less effective or more toxic alternatives [18].

Aminoglycosides, including gentamicin, amikacin, tobramycin, neomycin, and streptomycin, remain vital for treating serious Gram-negative infections [19]. Gentamicin is commonly used against *E. coli*, *K. pneumoniae*, *Serratia* spp., *Enterobacter* spp., and *Pseudomonas aeruginosa* [20]. It binds the 30S ribosomal subunit, disrupting protein synthesis and causing the production of truncated proteins that may be integrated into the cell wall, leading to cell impermeability, oxidative stress, and ultimately bacterial death [21,22]. Resistance to aminoglycosides arises through diverse mechanisms, including enzymatic drug modification, target site mutations, reduced uptake, and increased efflux [23].

Veterinary antibiotic use further complicates the AMR crisis. Florfenicol, a synthetic derivative of chloramphenicol, is widely employed in livestock and aquaculture for its broad-spectrum activity against respiratory and enteric infections. Like chloramphenicol, it inhibits protein synthesis by binding the 50S ribosomal subunit, and resistance commonly arises via antibiotic modification and efflux pumps [24,25]. Although florfenicol is not approved for human therapy, its use fosters cross-resistance and the transfer of ARGs to human pathogens, posing serious public health risks [26]. Moreover, agricultural application of antibiotics contributes substantially to the environmental ARG reservoir, facilitating dissemination through the food chain and other routes [27]. In this study, we include florfenicol as a veterinary benchmark to compare the evolutionary trajectories of chromosomal resistance loci across human and animal antibiotic exposures.

Clarifying how *E. coli* and other bacteria evolve resistance under varying antibiotic pressures is vital for guiding treatment and devising interventions to curb AMR. The MEGA-plate (Microbial Evolution and Growth Arena) experimental platform allows researchers to observe bacterial evolution in a spatially structured environment featuring gradient antibiotic concentrations [5], simulating real-world scenarios—clinical treatments, agricultural applications, and environmental exposures—where bacteria encounter fluctuating antibiotic levels. Previous MEGA-plate studies have documented the rapid emergence of resistance and the spatial dynamics of evolving bacterial populations. These works spotlight the roles of genetic mutation, horizontal gene transfer, and selective sweeps [5,24]. Nevertheless, comparative analyses of how diverse antibiotics shape resistance evolution in the same species are limited. Here, we incorporate genomic profiles from a published florfenicol evolution experiment chosen for its identical MEGA-plate design and sequencing pipeline. By benchmarking our meropenem and gentamicin results against these externally generated, but methodologically matched, florfenicol data, we can pinpoint cross-class resistome elements under truly comparative conditions.

This study aims to decipher the genotypic and phenotypic evolutionary trajectories of *E. coli* under the selective pressures of meropenem and gentamicin—key antibiotics in human medicine—and compare these results with florfenicol, a major veterinary antibiotic. By employing a modified MEGA-plate method, we seek to identify the mutational pathways, ARG dynamics, and resistance strategies that enable AMR and MDR. Our comparative approach will provide insights into how different antibiotic classes drive bacterial adaptation and resistance evolution, contributing to a broader understanding essential for designing effective strategies against AMR. While MEGA-plate evolution platforms have been used to visualize resistance emergence in single-drug contexts, our study innovates by systematically comparing two distinct clinical antibiotics (meropenem vs. gentamicin) alongside a veterinary benchmark (florfenicol data), thereby revealing both shared and antibiotic-specific adaptation pathways.

## 2. Results

### 2.1. Spatiotemporal Adaptation Dynamics

**Figure 1:**
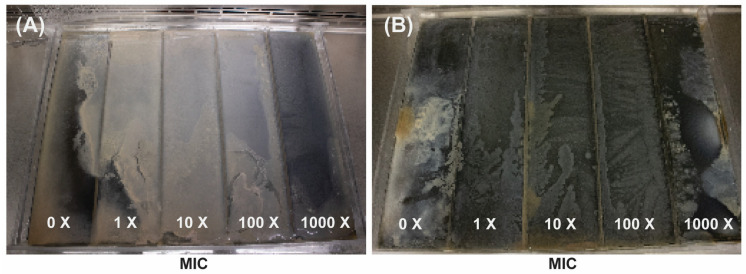
MEGA-plate experiments displaying bacterial colonization and adaptation across gradients of increasing antibiotic concentrations (0× to 1000× the minimum inhibitory concentration [MIC]) for meropenem (**A**) and gentamicin (**B**). (**A**) shows results from a 9-day incubation with meropenem, while (**B**) shows results from a 13-day incubation with gentamicin. Both images reveal how bacterial populations progressively adapt and spread into higher antibiotic concentrations over time.

Two independent *Escherichia coli* lineages were evolved on a modified MEGA-plate under meropenem and gentamicin selection, with assays running 9 and 13 days, respectively (Figure 1). Each plate was divided into five zones corresponding to 0×, 1×, 10×, 100×, and 1000× the minimum inhibitory concentration (MIC), set at 0.125 µg/mL for meropenem and 4 µg/mL for gentamicin. In the meropenem experiment (Figure 1A), all lineages traversed the gradient within 9 days, whereas gentamicin-exposed populations (Figure 1B) required the full 13 days to reach the highest concentration. Meropenem-selected fronts advanced rapidly through the gradient, whereas gentamicin-exposed populations progressed more slowly but with striking uniformity.

### 2.2. Variant Sampling and Sequencing

To define the genetic changes driving adaptation, we selected a total of eight meropenem-resistant and ten gentamicin-resistant isolates drawn across the five MIC zones (0×, 1×, 10×, 100×, 1000×). Genomic DNA from all eighteen isolates was deep-sequenced and mapped to the *Escherichia coli* ATCC 11229 reference. Variants were called using conservative filters (≥30× coverage; ≥75% allele frequency) and then annotated via the CARD’s RGI pipeline (≥90% identity/coverage). The CARD flagged only native chromosomal loci—no foreign contigs or mobile elements were detected—and from these loci we identified high-confidence mutations (Table 1).

### 2.3. Construction of ARG Databases

The CARD’s RGI pipeline identified 57 unique chromosomal resistance loci in meropenem-evolved genomes and 37 in gentamicin-evolved genomes (Supplementary Tables S1 and S2). These span RND (e.g., *acrAB–TolC*, *acrS*), MFS (e.g., *emrE*, *eptA*), SMR (*qacJ*), ABC (*msbA*) efflux families, global regulators (*marA*, *soxR*, *gadX*, *baeR*), two-component systems (*evgA/evgS*, *baeS*), and stress-response enzymes (*hns*, *vanG*). A complete breakdown of all CARD hits, mutation frequencies, and locus-by-antibiotic distributions is provided in the Supplementary Materials (Materials and Methods, Bioinformatic Analysis).

### 2.4. Distribution and Clustering of ARGs

**Figure 2:**
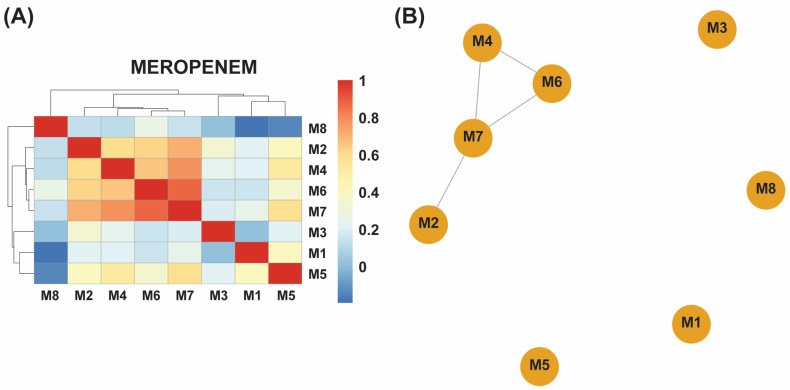
(**A**) A hierarchical clustering heatmap of antibiotic resistance genes (ARGs) detected in eight meropenem-exposed samples (M1–M8). The blue-to-red color scale represents the relative abundance or presence frequency of each ARG, revealing two distinct clusters: samples M2, M4, M6, and M7 share similar ARG profiles, whereas M1, M3, M5, and M8 display greater genetic variability. (**B**) An association network of the same samples further illustrates these relationships, with the closely connected cluster (M2, M4, M6, and M7) reflecting similar ARG compositions and the more isolated samples (M1, M3, M5, and M8) indicating higher ARG diversity in response to meropenem exposure.

**Figure 3:**
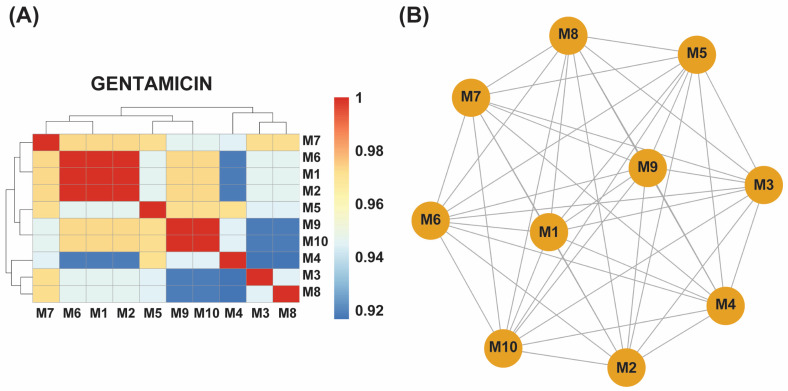
(**A**) A hierarchical clustering heatmap showing antibiotic resistance genes (ARGs) detected in ten gentamicin-exposed samples (M1–M10). The blue-to-red color scale indicates the relative abundance or presence frequency of each ARG. Samples M1, M2, and M6 form a distinct cluster with identical ARG profiles, while M9 and M10 exhibit another shared cluster. Other samples (M3, M4, M5, M7, and M8) correlate closely with these clusters, indicating a more uniform ARG distribution in response to gentamicin exposure. (**B**) An association network of the gentamicin-exposed samples further illustrates the relationships among their ARG profiles. M1, M2, and M6 are highly interconnected, reflecting identical ARG profiles, while M9 and M10 are similarly grouped. The remaining samples show a strong correlation with these clusters, confirming a more uniform resistance response across all ten samples.

Hierarchical clustering of presence–absence profiles revealed two clear subgroups in each dataset (Figure 2A,B and Figure 3A,B): a core cluster enriched for efflux regulators and SOS-linked loci (e.g., *acrS*, *baeR*, *hns*) versus a more variable cluster carrying additional stress-response or inactivation genes. In the meropenem dataset, the core cluster comprises M2, M4, M6, and M7, while M1, M3, M5, and M8 form the divergent group; gentamicin lineages split similarly around their shared efflux-centric resistome. Notably, *acrS*, *eptA*, *emrE*, *evgA*, *hns*, and *vanG* were conserved across all eight meropenem-evolved samples, while *leuO* and the locus annotated as EC-13 appeared exclusively in M1, and *uhpT* was uniquely retained in M8 (Supplementary Table S1).

By contrast, gentamicin-evolved isolates displayed greater homogeneity. Three samples (M1, M2, M6) shared identical locus profiles, and another pair (M9, M10) likewise clustered together, with the remaining five (M3, M4, M5, M7, M8) showing high similarity to these core groups. Across all ten gentamicin-evolved samples, *marA*, *gadX*, and *hns* remained ubiquitous, while *uhpT* was present in six isolates (M1, M2, M3, M6, M7, M8), and *mdtF* appeared in five (M1, M2, M6, M9, M10) (Supplementary Table S2).

These clustering patterns underscore a heterogeneous adaptive response to meropenem—reflected by distinct subgroups with variable locus repertoires—versus a more unified response to gentamicin, in which resistance-associated loci were broadly shared across evolved populations.

### 2.5. Resistance Mechanisms Identified

Efflux pump systems emerged as the predominant resistance mechanism in both datasets, facilitating active extrusion of antibiotics and reducing intracellular drug concentrations. Several efflux pump families were identified: Resistance-Nodulation-Division (RND), Major Facilitator Superfamily (MFS), Small Multidrug Resistance (SMR), and ATP-Binding Cassette (ABC) transporters. 

In the meropenem-evolved isolates, the CARD annotation revealed a broad spectrum of efflux and accessory systems. Within the RND family, we observed frequent hits to *acrB*, *acrD*, *acrE*, *acrF*, *acrS*, and *adeF*, alongside the complete AcrAB–TolC complex with mutations in its regulators *acrR* and *marR*. The two-component regulator *baeR*, the envelope-stress sensor *cpxA*, and the global transcriptional regulator *crp* were also repeatedly flagged. Downstream modulators *gadX*, *marA*, and the small RNA chaperone *rsmA* featured prominently, as did multiple subunits of the Mex-type pumps—*mdtA*, *mdtB*, *mdtC*, *mdtE*, *mdtF*—and the membrane channel *tolC* itself.

MFS-family pumps were equally well represented, with hits to *emrA*, *emrB*, *emrK*, *emrR*, *mdfA*, *mdtG*, *mdtH*, *mdtN*, *mdtO*, *mdtP*, and the transcriptional regulator *leuO*. SMR-family loci included the native *emrE*, the CARD-annotated *kpnF* homolog, and *qacJ*. From the ABC family, only *msbA* appeared (in addition to *tolC* under RND annotation), and no MATE-family genes were detected.

Beyond efflux, loci involved in target modification and stress response were prominent: *arnT*, *bacA*, *eptA*, the elongation factor *tuf* (EF-Tu), the hexose phosphate transporter *uhpT*, and the vancomycin-related ligase *vanG*. Two inactivation-related genes—*ampC* β-lactamase and the uncharacterized locus *EC-13*—were identified, and the KdpDE two-component kinase *kdpE* was found in all but one lineage (M8) (Supplementary Table S1).

In the gentamicin-evolved isolates, resistance again centered on RND-family efflux pumps, with the CARD annotation flagging *acrS*, *adeF*, and the AcrAB–TolC complex bearing *marR* mutations. Two-component regulators *baeR* and *baeS*, the global regulator *crp*, and master transcription factors *gadX* and *marA* were also consistently detected, as were the RND-linked modulators *mdtE*, *mdtF*, and the small RNA chaperone *rsmA*. Notably, the CARD additionally flagged a homolog of *acrA*.

MFS-family pumps featured prominently, with hits to *emrB*, *emrK*, *emrR*, *emrY*, *leuO*, *mdtM*, *mdtN*, *mdtO*, and *mdtP*. SMR-family loci included the native *emrE*, alongside *kpnE* and *kpnF* homologs and *qacJ*. From the ABC transporter family, only *msbA* appeared. Unlike meropenem-evolved lines, no single gentamicin isolate co-flagged ABC, MFS, and RND pumps simultaneously; however, the two-component pair *evgA/evgS* and the nucleoid-associated protein *hns* bridged efflux and regulatory networks across multiple isolates. Inactivation mechanisms were limited to a single β-lactamase variant (*act-17*).

These patterns mirror those seen under meropenem selection—with a dominant role for RND and MFS efflux systems complemented by global regulators and stress-response loci—but they exhibit a more uniform distribution of resistance determinants and a narrower repertoire of inactivation genes (Supplementary Table S2).

### 2.6. Single Nucleotide Polymorphisms (SNPs) and Mutations

High-confidence variant calling identified a handful of recurrent mutations in both antibiotic settings (Supplementary Tables S1 and S2). In meropenem-evolved isolates, two missense mutations—Y137H and G103S—were detected in the *marR* regulator of the AcrAB–TolC pump, likely modulating efflux expression. Additional nonsynonymous changes included *tufA* (encoding EF-Tu) R234F, *glpT* E448K, *uhpT* E350Q, and dual substitutions in the penicillin-binding protein *pbp3* (D350N, S357N). Gentamicin-evolved populations converged on three of these loci, with EF-Tu R234F, UhpT E350Q, and PBP3 D350N/S357N recurring across independent lineages. These alterations—found in transporters, global regulators, and target enzymes—are poised to affect drug uptake, binding affinity, and efflux dynamics, collectively contributing to elevated resistance.

### 2.7. Resistance to Disinfectants and Antiseptics

In addition to antibiotic-specific determinants, both meropenem- and gentamicin-evolved populations carried chromosomal loci previously associated with tolerance to disinfectants and antiseptics. Meropenem-selected isolates uniformly flagged the outer-membrane channel *tolC*, the regulator *marA*, and the RND-pump subunits *acrA*, *acrB*, and *acrS* together with mutations in *acrR* and *marR*. Several MFS pumps (*mdtN*, *mdtO*, *mdtP*, *mdtM*, *mdfA*), the phosphoethanolamine transferase *eptA*, and the SMR-family transporter *qacJ* were also detected, as were stress-response regulators *leuO*, *soxR*, and *soxS*. Homologs annotated by the CARD as *kpnE* and *kpnF* likewise appeared, reflecting *E. coli* variants of these efflux components (Supplementary Table S1).

Gentamicin-evolved isolates exhibited a similar antiseptic-tolerance profile. The RND pumps *AcrS* and *TolC* (with *MarR* mutations), the MFS pumps *MdtN*, *MdtO*, and *MdtP*, and the SMR transporter *QacJ* were consistently present, alongside *marA*, *leuO*, and the two-component regulators *BaeR/BaeS*. The CARD-labeled homologs *kpnE* and *kpnF* appeared again, and an *acrA* homolog was flagged in several lines (Supplementary Table S2).

The co-occurrence of antibiotic- and biocide-tolerant loci underscores the potential for cross-resistance. Enrichment of these efflux and regulatory systems may compromise disinfection protocols in clinical and agricultural settings, facilitating the persistence and spread of resistant *E. coli*.

### 2.8. Phenotype of Resistant Strains

**Figure 4:**
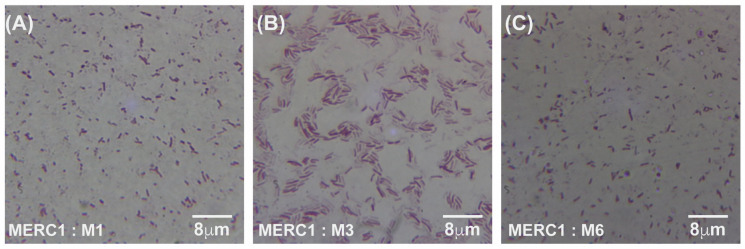
Gram-stained micrographs (100× magnification) of *E. coli* samples taken from the meropenem control lane (C1), which contained no antibiotic. Panels (**A**–**C**) correspond to samples M1, M3, and M6, respectively, all originating from the same antibiotic-free lane. MER = meropenem, C1 = lane without antibiotic, and M = sample number from that lane.

**Figure 5:**
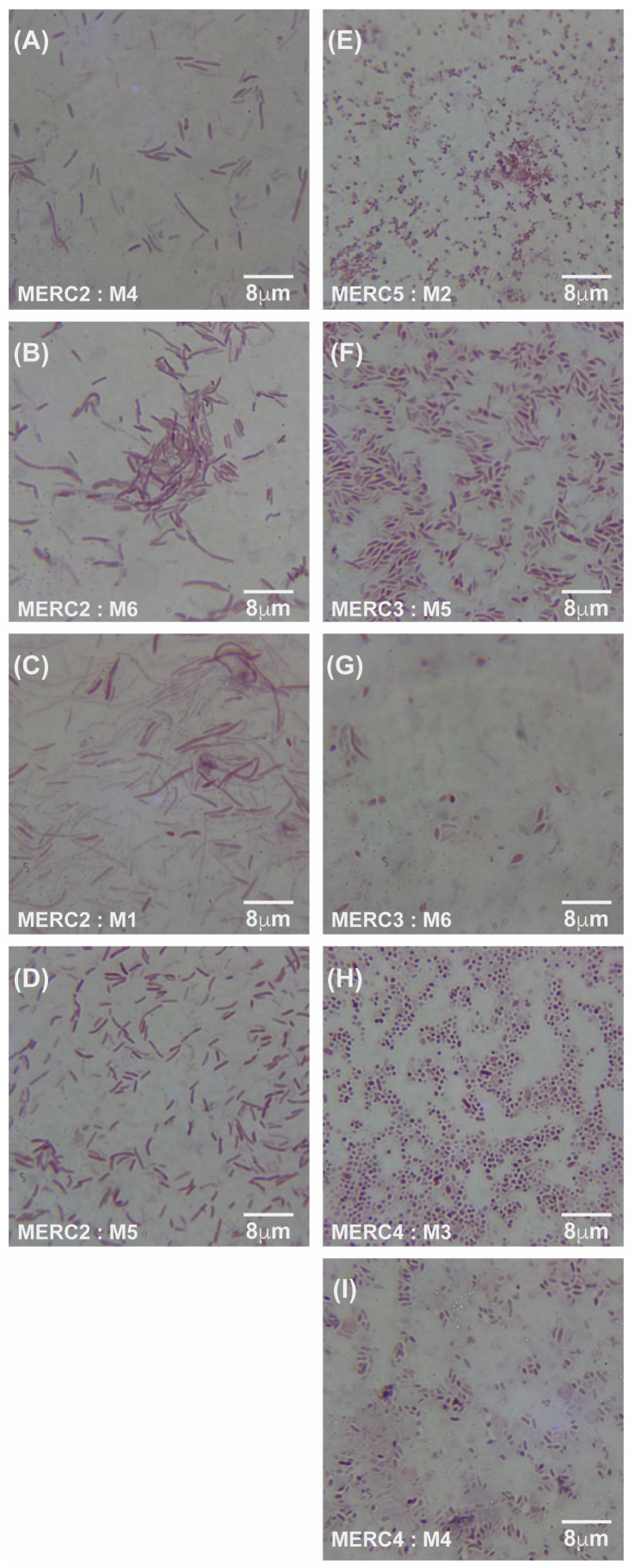
Gram-stained micrographs (100× magnification) of *E. coli* samples. (**A**–**D**) display samples from meropenem lane 2 (C2) at an MIC of 0.125 μg/mL, with corresponding micrographs of samples M4, M6, M1, and M5, respectively. (**E**–**I**) show samples from lanes 3, 4, and 5 (C3–C5), with micrographs of M2 from lane 5 at an MIC of 125 μg/mL, M5 and M6 from lane 3 at an MIC of 1.25 μg/mL, and M3 and M4 from lane 4 at an MIC of 12.5 μg/mL. MER = meropenem, C2–C5 = lane numbers, and M = sample number from the respective lane.

**Figure 6:**
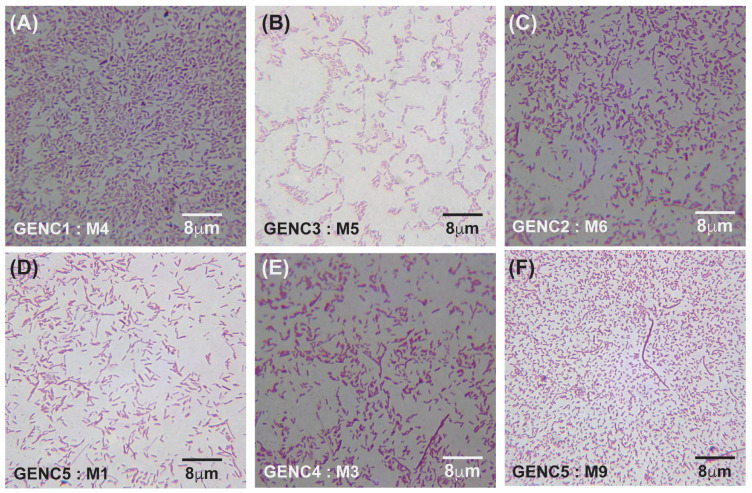
Gram-stained micrographs (100× magnification) of *E. coli* samples obtained from gentamicin-treated lanes. (**A**–**C**) represent samples from lanes 1 (C1), 2 (C2), and 3 (C3): panel (**A**) corresponds to sample M4 from antibiotic-free lane 1, panel (**B**) to sample M5 from lane 3 at an MIC of 40 μg/mL, and panel (**C**) to sample M6 from lane 2 at an MIC of 4 μg/mL. (**D**–**F**) show samples from lanes 4 (C4) and 5 (C5): panel (**D**) represents sample M1 from lane 5 at an MIC of 4000 μg/mL, panel (**E**) represents sample M3 from lane 4 at an MIC of 400 μg/mL, and panel (**F**) represents sample M9 from lane 5 at an MIC of 4000 μg/mL. GEN denotes gentamicin, C indicates the lane number, and M refers to the sample number from each lane.

Under meropenem, *E. coli* progressed from rods (0× MIC) to elongated filaments (1×–10× MIC) and, at the highest concentrations (100×–1000× MIC), to small spheroplast-like cells, a pattern consistent with SOS-driven division arrest and cell-wall remodeling (Figure 4A–C and Figure 5A–I). Gentamicin-exposed populations retained predominantly rod shapes, showing only moderate elongation at mid-MIC zones (Figure 6A–F). A more comprehensive description of Figure 4, Figure 5 and Figure 6 can be found in the Supplementary Materials and Methods Section Selection of Resistant Phenotypes.

## 3. Comparative Analysis of Meropenem, Gentamicin, and Florfenicol Resistance Profiles

To identify both common and antibiotic-specific adaptations, we compared our meropenem- and gentamicin-evolved *E. coli* loci with those reported for florfenicol-selected populations by Kerek et al. (2024) [24]. Across the three datasets, we catalogued 64 unique resistance-associated loci, of which 22 were shared under all three selection pressures (Table 1).

The shared loci—*acrS*, *baeR*, *crp*, *emrB*, *emrK*, *emrR*, *emrY*, *eptA*, *emrE*, *evgA*, *evgS*, *gadX*, *hns*, *marA*, *mdtE*, *mdtF*, *mdtM*, *mdtN*, *mdtO*, *mdtP*, *msbA*, and *pmrF*—encode core components of multidrug efflux systems (RND and MFS families), global regulators of stress response and pump expression, and enzymes that alter the cell envelope. Their consistent detection across carbapenem, aminoglycoside, and phenicol challenges highlights a universal chromosomal network that *E. coli* recruits to tolerate diverse antibiotics.

Each antibiotic regimen also selected for unique loci reflecting specialized mechanisms. Under florfenicol, *ampH*, *gadW*, and *ugd* appeared exclusively; *ampH* encodes a class C β-lactamase, *GadW* regulates acid-response genes, and *Ugd* modifies lipopolysaccharide structure, potentially reducing drug influx. In the meropenem lines, unique hits included the uncharacterized locus *EC-13*, the efflux repressor *acrR* (mutated within the AcrAB–TolC complex), the glycerol-3-phosphate transporter *glpT*, and the oxidative-stress regulators *soxR* and *soxS*. These mutations likely drive the overexpression of efflux or stress-defense pathways specific to β-lactam pressure. Gentamicin selection yielded two bespoke loci: the β-lactamase variant *act-17* and an *acrA* homolog, suggesting possible co-selection or structural variations in the efflux apparatus under ribosomal-targeting stress.

Together, these findings reveal a dual strategy of adaptation in *E. coli*: a shared core resistome of regulatory and efflux functions that underpins multidrug tolerance, and antibiotic-specific loci that fine-tune the response to distinct mechanisms of action. The identification of common loci across all three drug classes highlights potential broad-spectrum targets, while the unique adaptations associated with each antibiotic indicate specialized mechanisms that may be critical targets for antibiotic development and therapeutic interventions.

## 4. Functional Categorization of Antibiotic Resistance Genes

**Figure 7:**
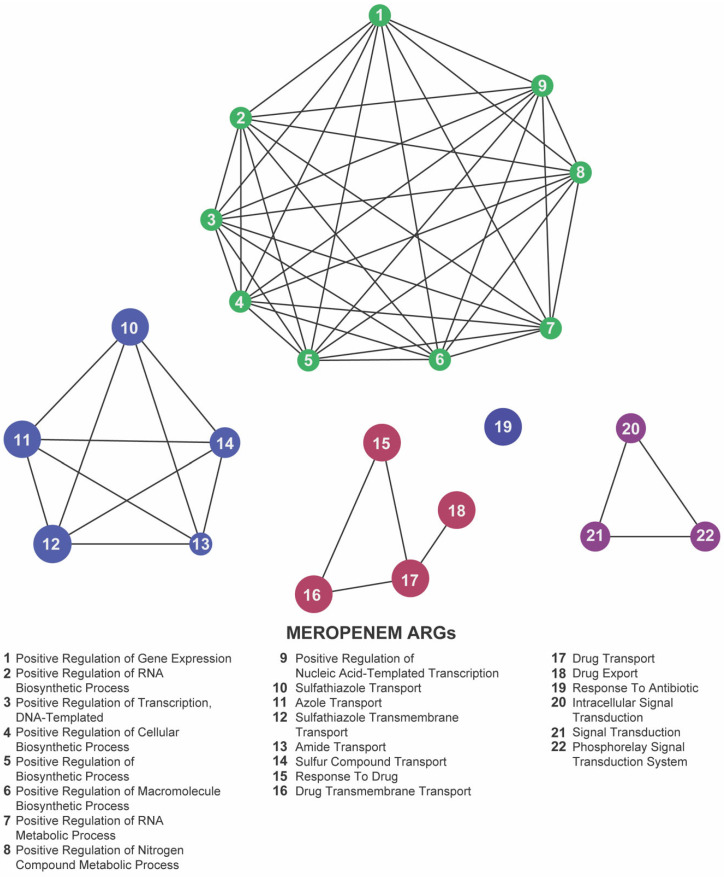
Network representation of biological functions associated with meropenem-related antibiotic resistance genes (ARGs). Each numbered node corresponds to a specific function (listed below), and the edges indicate relationships or co-occurrence between these functions. The clusters of nodes (indicated by distinct colors) represent groups of related processes—regulation of gene expression, biosynthetic activities, and antibiotic transport or response mechanisms—highlighting the complex functional landscape that emerges under meropenem pressure.

**Figure 8:**
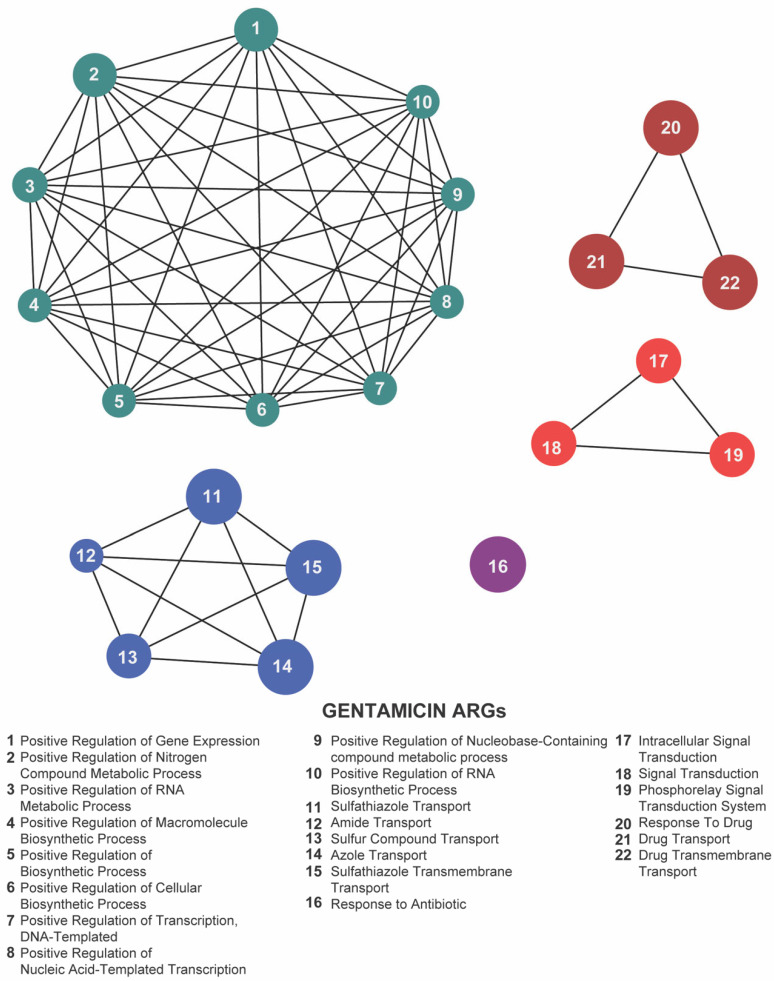
Network representation of biological functions associated with gentamicin-related antibiotic resistance genes (ARGs). Each numbered node corresponds to a specific function (listed below), and the edges indicate relationships or co-occurrence among these functions. The clusters of nodes (color-coded) represent interconnected groups of biological processes—metabolic regulation, biosynthetic pathways, and antibiotic transport or response mechanisms—highlighting a tightly coordinated functional network under gentamicin exposure.

To uncover the overarching biological processes underpinning resistance, we performed gene ontology (GO) enrichment analysis using the ClueGO plugin. In both our meropenem- and gentamicin-evolved datasets, five GO terms were significantly overrepresented: positive regulation of gene expression, sulfathiazole transport, response to drug, response to antibiotic, and intracellular signal transduction (Figure 7 and Figure 8; Supplementary Tables S3 and S4). These categories emphasize the coordinated engagement of transcriptional regulators, efflux systems, and signaling pathways as *E. coli* adapts to antibiotic stress.

**Figure 9:**
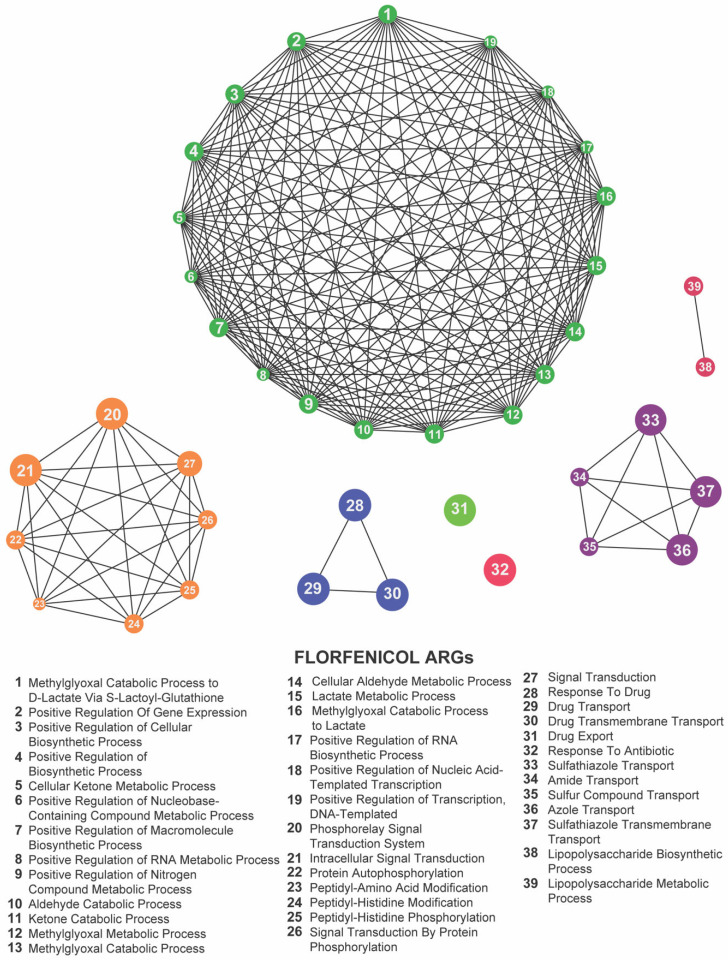
Network representation of biological functions associated with florfenicol-related antibiotic resistance genes (ARGs). Each numbered node corresponds to a specific function (listed below), and the edges indicate relationships or co-occurrence among these functions. The large, densely connected cluster represents a complex network of metabolic and biosynthetic processes, while the smaller groups highlight additional functions such as protein modifications, signal transduction pathways, and antibiotic transport or response. Together, these clusters illustrate the intricate functional landscape influenced by florfenicol exposure.

By contrast, florfenicol-resistant populations (Kerek et al., 2024) [24] exhibited enrichment in seven GO terms: methylglyoxal catabolic process to D-lactate via S-lactoyl-glutathione, lipopolysaccharide metabolic process, drug export, response to antibiotic, response to drug, phosphorelay signal transduction system, and sulfathiazole transport (Figure 9; Supplementary Table S5). The prominence of metabolic pathways—particularly the methylglyoxal detoxification and LPS remodeling—suggests that florfenicol selection uniquely pressures *E. coli* to rewire its metabolism alongside efflux and regulatory responses.

**Figure 10:**
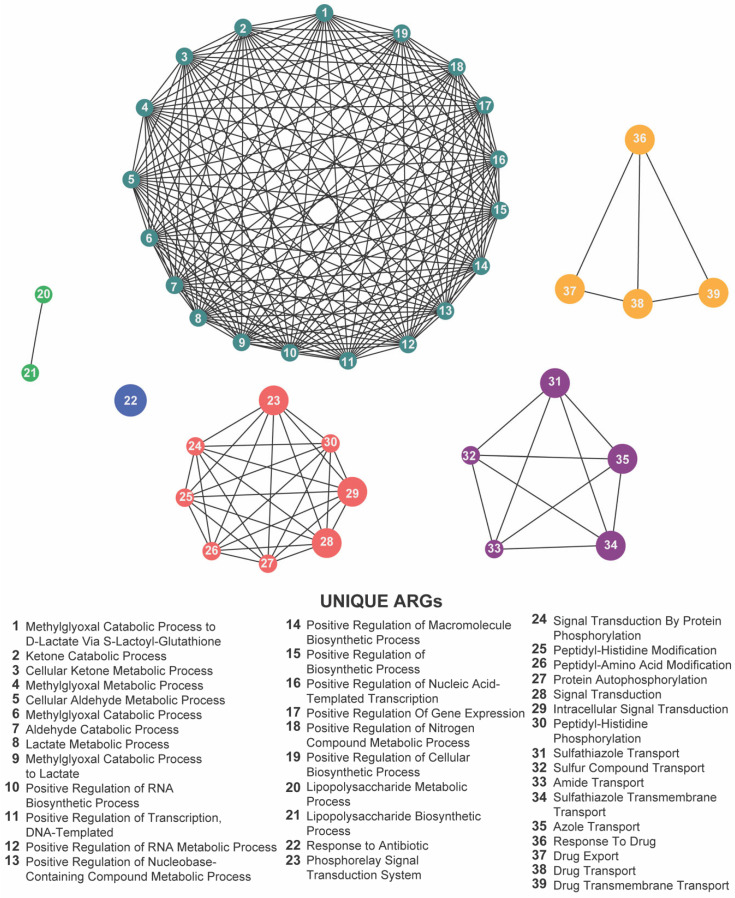
Network visualization of distinct biological functions associated with antibiotic resistance genes (ARGs) identified under exposure to meropenem, gentamicin, and florfenicol. Each numbered node represents a unique biological function (listed below), and the edges indicate functional relationships or co-occurrences. The densely interconnected cluster, along with the multiple smaller groups, highlights a wide range of metabolic, biosynthetic, regulatory, and transport-related processes that collectively shape the complex resistance landscape across these antibiotics.

Focusing on the 22 loci shared across the meropenem, gentamicin, and florfenicol lines, six GO terms remained significantly enriched: methylglyoxal catabolic process to D-lactate via S-lactoyl-glutathione, lipopolysaccharide metabolic process, response to antibiotic, response to drug, phosphorelay signal transduction system, and sulfathiazole transport (Figure 10; Supplementary Table S6). The recurrence of these processes highlights a core functional network in which *E. coli* integrates metabolic flexibility with sophisticated regulatory circuits and efflux-mediated transport to survive diverse antibiotic pressures.

Together, these enrichment profiles underscore the multifaceted nature of chromosomal adaptation: while gene-expression regulation and intracellular signaling form the backbone of a shared resistance strategy, drug-specific metabolic pathways further refine the response to individual antibiotic classes. These findings provide valuable insights into the biological functions associated with resistance-associated loci and highlight potential targets for novel antimicrobial strategies.

## 5. Discussion

### 5.1. Genomic Adaptations

By applying the MEGA-plate system, we tracked the real-time adaptation of *Escherichia coli* under meropenem and gentamicin gradients [5] and benchmarked these dynamics against florfenicol-evolved strains [24].

Meropenem selection yielded highly heterogeneous resistance profiles: four isolates (M1, M3, M5, M8) diverged sharply from the other half of the population. This pattern implies that multiple mutational pathways can confer survival at elevated meropenem concentrations, reflecting the interplay of mutation, selection, and clonal competition. Spontaneous mutants with variable fitness [28] arise under antibiotic stress [29,30]. Baym et al. (2016) [5] showed that, under trimethoprim, faster-growing but less resistant clones can overtake hyper-resistant mutants. Our observation of coexisting, divergent meropenem lineages aligns with these competitive dynamics.

By contrast, gentamicin-exposed populations—and florfenicol lines from Kerek et al. (2024) [24]—showed uniform adaptive responses. This conserved response suggests reliance on a core efflux-and-regulator toolkit when facing aminoglycosides or phenicols.

Within meropenem-adapted populations, six SOS-linked loci—*acrS*, *eptA*, *emrE*, *evgA*, *hns*, and *vanG*—were omnipresent, marking them as central hubs of resistance. AcrS represses AcrAB–TolC efflux [31]; EptA modifies lipid A to deter cationic peptides [32]; EmrE exports diverse toxins [33]; EvgA regulates acid-stress and efflux genes [34,35]; H-NS globally silences or activates stress loci [36,37]; and VanG—in spite of its enterococcal origin—may reflect chromosomal remodeling [38].

The enrichment of H-NS under all three antibiotics underscores its versatility as a global repressor, modulating virulence, stress, metabolism, and foreign gene expression [39,40]. Its recurrent detection suggests that H-NS helps synchronize multi-drug stress responses.

Although the gentamicin and florfenicol lines maintained their core ARG sets, we observed the variable presence of *uhpT*—a hexose phosphate importer regulated by UhpABC [41,42,43]—and of *mdtF*, an MdtEF–TolC efflux component [44,45]. These fluctuations imply the modulation of nutrient uptake and pump expression under aminoglycoside stress.

Both meropenem and gentamicin regimens co-select loci known to mediate biocide tolerance—*qacJ*, *mdfA*, *mdtM*, and AcrAB–TolC [46,47,48]. Such cross-resistance threatens sterilization efficacy and underscores the need to regulate biocide usage in healthcare settings.

Our screen did not detect any plasmid-borne or other mobile-element–associated resistance factors; all hits mapped to the chromosome. This likely reflects both the starting strain and the relatively short timescale and controlled conditions of the MEGA-plate experiment, which may not favor horizontal transfer of large resistance plasmids. In more complex or longer-term settings—where bacteria interact with environmental reservoirs or other species—plasmid-mediated and transposon-associated ARGs often play a major role. Thus, while our chromosomal focus has illuminated the core, endogenous resistome recruited under steep gradients, it may underestimate the full spectrum of resistance strategies available in natural settings.

While our genomic and phenotypic data nominate master regulators (e.g., *marA*, *baeR*) and efflux loci (e.g., *acrS*, *mdtE*) as key resistance nodes, direct confirmation via gene knock-out or overexpression remains to be conducted. In future work, we plan the targeted deletion of *marA* and *abeR* and heterologous overexpression of *acrS* in wild-type *E. coli* to quantify their individual contributions to MIC shifts. These experiments will validate the causal roles of the core resistome uncovered here.

### 5.2. Phenotype of Resistant Strains

Beyond the gene-level shifts, we also observed striking, antibiotic-specific cell-shape changes. Meropenem targets penicillin-binding proteins (PBPs), disrupting cell-wall synthesis [49,50,51]. Under such stress, *Enterobacteriaceae* trigger SOS-linked loci [52], form cell-wall–deficient spheroplasts [53], alter division machinery, rewire envelope biogenesis and ATP metabolism [51], and generate persisters [54,55]—all of which echo the diverse mechanisms flagged in our meropenem-adapted genomes.

Gentamicin binds the 30S ribosomal subunit, causing mistranslation and ribosome stalling [19]. Resistance chiefly involves antibiotic-modifying enzymes, efflux pumps, and ribosomal mutations [23]. Although SOS activation and major shape changes are uncommon under aminoglycosides, cell elongation and reduced ribosome density have been noted [56], mirroring our gentamicin lines’ uniform ARG set and modest morphological shifts.

Morphological adaptations under antibiotic stress can influence both survival in clinical reservoirs [57,58,59] and the efficacy of disinfection protocols [60,61,62]. In our MEGA-plate experiments, the divergent ARG repertoires under meropenem contrasted sharply with the uniform ARG set under gentamicin—and these genetic patterns tracked with equally divergent morphological outcomes.

Under meropenem, cells progressed from rods (0× MIC) to filaments at mid-MIC—indicative of SOS-induced division arrest—and then through oval intermediates to coccoid forms at the highest concentrations, echoing β-lactam–induced spheroplast-like states. Similar to ciprofloxacin-induced filamentation, meropenem triggers patterns that activate SOS-mediated mutagenesis [52], generating elongated cells that can yield genetically diverse progeny [52].

These divergent morphologies—dramatic remodeling under cell-wall stress versus conservative shape under ribosomal stress—underscore how the antibiotic mode of action dictates the phenotypic pathways of adaptation.

Moreover, the circular morphology observed at the highest antibiotic concentrations does not correspond to previously described spheroplasts, indicating the possibility of an uncharacterized survival strategy that warrants further investigation. In addition, the stability of ARG profiles and the morphological changes in gentamicin-exposed samples in the last lanes in *E. coli* suggest a different adaptive approach. The lack of significant phenotypic alterations may reflect a reliance on inherent resistance mechanisms, such as efflux pumps or enzymatic modifications, rather than inducing extensive morphological transformations.

By imposing spatial antibiotic gradients, the MEGA-plate system captures both mutation and clonal competition in real time. In our hands, it uniquely revealed the dichotomy between multi-pathway meropenem adaptation and uniform gentamicin resistance—insights that would be obscured in well-mixed or static assays.

Our cross-drug comparison [63] pinpointed a core resistome of 22 chromosomal loci shared evenly with florfenicol-evolved strains. Recognizing such universal nodes—rather than isolated ARGs—will sharpen both drug-development efforts and molecular surveillance pipelines.

Identifying a shared core resistome [64,65,66] of 22 loci across human (meropenem, gentamicin) and veterinary (florfenicol) antibiotics reinforces a One Health framework [67,68]. Agricultural antibiotic use can seed environmental ARGs that eventually infiltrate clinical strains or the food chain [27,69], highlighting the necessity of coordinated stewardship.

Future research should expand on challenging *E. coli* with antibiotic pairs (e.g., meropenem plus efflux pump inhibitor) on MEGA-plates to dissect synergistic or antagonistic trajectories. Such combinatorial gradients may reveal collateral sensitivities exploitable in therapy.

We demonstrate that mechanism-specific evolutionary routes—morphological remodeling under meropenem vs. efflux stability under gentamicin—coexist atop a shared chromosomal backbone of resistance loci. Integrating these insights into One Health frameworks will be essential for next-generation antimicrobial design and stewardship.

### 5.3. Biological Functions

GO enrichment highlighted the positive regulation of gene expression as the top functional category in both meropenem- and gentamicin-adapted populations, driven primarily by three master regulators: *baeR*, *gadX*, and *marA*.

BaeR (of the BaeSR two-component system) [70,71] upregulates key efflux pumps (e.g., *mdtABC*, *acrD*) to export diverse toxins—β-lactams, novobiocin, detergents, and bile salts [34,72]. It also controls a broader regulon (>50 genes) spanning signal transduction and membrane biogenesis [72]. Its GO enrichment in our dataset underscores BaeR’s central role in coordinating efflux-based and envelope-related defenses against antibiotic stress.

GadX drives the glutamate-dependent acid-resistance system while also modulating sugar catabolism and general stress-response genes [73,74,75]. Its enrichment here suggests *E. coli* co-opts acid-stress circuitry to bolster survival during antibiotic challenge.

MarA orchestrates the mar regulon [75], boosting AcrAB–TolC efflux, downregulating porins (e.g., *ompF*), and upregulating oxidative-stress defenses [76,77]. This multifaceted control reduces intracellular drug levels and enhances survival under diverse antibiotic stresses.

Collectively, the GO enrichment of *baeR*, *gadX*, and *marA* highlights transcriptional regulation as a linchpin of adaptation—driving efflux, membrane remodeling, and stress responses in concert. Disrupting these regulators may therefore cripple multiple resistance routes simultaneously.

Standard MIC and disk-diffusion tests overlook dynamic factors [78]—population density, biofilms, sub-MIC zones, and ecological interactions—that drive real-world resistance evolution [30,79,80,81,82]. Furthermore, it is increasingly recognized that microbial susceptibility to antibiotics is not solely an intrinsic property but is significantly influenced by interspecies interactions and ecological niche conditions [83,84]. The microbial community context can modulate gene expression, horizontal gene transfer, and metabolic cooperation or competition, all of which impact the emergence and spread of AMR.

By imposing spatially graded antibiotic pressures, the MEGA-plate captures in situ mutation, selection, and competition—revealing both heterogeneous and uniform adaptive strategies that static assays miss.

Therefore, here we show that *E. coli* deploys mechanism-tailored adaptations—extensive remodeling under meropenem versus efflux-centric stability under gentamicin—anchored by a shared set of transcriptional regulators. Targeting these regulatory nodes offers a promising path to disrupt multifaceted resistance and inform next-generation antimicrobial design.

### 5.4. Study Limitations—Contextual Considerations

Our study has several key constraints that should inform the interpretation and generalizability of these findings. First, the MEGA-plate experiments ran for only 9–13 days and employed a single laboratory strain of *E. coli*, likely underrepresenting the full diversity of resistance trajectories accessible to clinical or environmental isolates in polymicrobial contexts. Second, by design, we focused exclusively on chromosomal mutations and did not capture plasmid-, transposon-, or integron-mediated horizontal gene transfer, which plays a major role in resistance dissemination in natural and clinical settings. Third, although nanopore sequencing provided broad coverage of point mutations and gene presence, it may have missed low-frequency variants, structural rearrangements, and small indels; deeper, longer-read, or complementary short-read sequencing would improve detection of these events. Fourth, our modifications to the standard Baym et al. [5] MEGA-plate—downsizing to 40 × 50 cm and adapting for orbital incubator and laminar-hood constraints—optimized handling and temperature uniformity but could subtly affect gradient diffusion dynamics; while we observed no obvious impact on bacterial migration or resistance emergence, quantitative comparisons to the original design remain to be made. Finally, we did not experimentally validate the phenotypic effects of the identified resistance loci; future work using gene knock-outs, overexpression assays, and fitness measurements in diverse strains will be critical to confirm their causal roles and assess their clinical relevance. Together, these limitations highlight the need for extended evolution experiments, inclusion of multiple strains and resistance mechanisms, and targeted functional studies to build on the chromosomal resistome framework presented here.

## 6. Materials and Methods

### 6.1. Microbial Strains and Conditions

*Escherichia coli* ATCC 11229 was used as the model organism. Cultures were maintained in Luria–Bertani (LB) broth (Difco Laboratories, Detroit, MI, USA) at 37 °C with 150 rpm agitation. Overnight cultures were initiated from single colonies and grown for 16 h. See Supplementary Materials for full details.

### 6.2. Determination of Minimum Inhibitory Concentration (MIC)

MICs for meropenem and gentamicin were determined via broth microdilution following adapted CLSI guidelines [85,86]. Stock solutions (prepared per manufacturers’ instructions) were used to achieve initial concentrations of 1 µg/mL (meropenem) and 32 µg/mL (gentamicin) in 200 μL volumes. Serial dilutions (1–0.0625 µg/mL for meropenem and 16–0.5 µg/mL for gentamicin) were performed. An overnight culture was adjusted to an OD_600_ of 0.2 (~10^7^–10^8^ cells/mL) and diluted 1:100, and 100 μL was added to each well (final concentration ≈10^5^ cells/mL). Plates were incubated at 37 °C for 24 h, with MIC defined as OD_600_ < 0.05. All experiments were run in triplicate. See Supplementary Materials for complete protocols.

### 6.3. Design of the 40 × 54 cm Acrylic MEGA-Plate

A modified acrylic plate was constructed to study bacterial migration and resistance emergence (Supplementary Figure S1), adapted from Baym (2016) [5].

To fit our incubator (IS-971/IL-21A) and laminar-flow hood, we scaled the plate down to 40 × 50 cm acrylic, glued with dichloromethane and sealed with silicone to prevent leakage. Three agar layers (contrast base, support middle, 0.28% surface) were cast sequentially to preserve steep 0×–1000× MIC gradients, and the entire assembly was designed for sterile manipulation and uniform 37 °C incubation. These adaptations maintain the original diffusive-growth dynamics while improving handling and contamination control. For construction details and dimensions, refer to the Supplementary Materials.

### 6.4. Sterilization of the MEGA-Plate and Culture Media

Prior to use, each antibiotic-specific plate was disinfected overnight with 5–10% diluted hypochlorite. Six liters of LB medium underwent a double sterilization cycle and were distributed in three layers (base, intermediate, and surface) with varying agar concentrations. The base layer (2 L) contained 2% agar and 30 µg/mL kanamycin; both the base and intermediate layers included India ink (4 mL). The surface layer was prepared as a semi-solid medium (0.28% agar) with an antibiotic gradient (0, 1×, 10×, 100×, and 1000× MIC). Additional specifics are in the Supplementary Materials.

### 6.5. Inoculation of E. coli Bacteria and Selection of Resistant Phenotypes

A 300 μL aliquot of the standardized culture was inoculated along the edge of the antibiotic-free lane using a sterile pipette tip to form a uniform starting line. Plates were incubated at 37 °C for 9–13 days, allowing migration across the gradient. Colonies appearing beyond the initial MIC zone were deemed resistant. Six representative colonies per lane were subcultured on LB agar with the corresponding antibiotic concentration and in liquid LB broth (triplicate cultures), then stored at −80 °C in LB with 20% glycerol. See Supplementary Materials for detailed selection and confirmation procedures.

### 6.6. DNA Extraction, Sequencing, and Bioinformatic Analysis

Genomic DNA from selected meropenem- and gentamicin-resistant isolates was extracted using the PureLink Genomic DNA Mini Kit (Invitrogen, Carlsbad, CA, USA) and sequenced via nanopore technology at Secoya Labs [87]. Sequences were analyzed using the Comprehensive Antibiotic Resistance Database (CARD) (https://card.mcmaster.ca/, accessed on 22 June 2025) [88] through the RGI platform with strict thresholds (≥90% identity/coverage) to identify resistance genes [89,90,91]. Subsequent analysis included constructing heat maps, network visualizations using R (version 6.0.5) [92] Cytoscape (version 3.10.3) [93] and the ClueGo (version 2.5.1) gene ontology enrichment plugin [94], and hierarchical clustering based on the Jaccard similarity index [95] to compare gene profiles across antibiotic concentrations. A heatmap was created using color-coded genes (blue for absence and red for presence) to visually represent gene presence across the antibiotics [96]. A bar plot was then utilized to illustrate the total number of genes present for each antibiotic [97], followed by a stacked bar plot to depict the proportion of genes present or absent for each antibiotic. For network analyses, pairwise binary similarity between isolates in the gentamicin dataset was calculated using the Jaccard index. Similarity matrices were thresholded at 0.40, which was chosen after evaluating values between 0.30 and 0.50 for interpretability and connectivity balance. For the meropenem dataset, Pearson correlation coefficients were computed between binary gene-presence profiles, and a threshold of 0.65 was applied after testing values from 0.35 to 0.65; this value preserved meaningful relationships while avoiding spurious edges. Threshold selection was based on the distribution of similarity values, a visual inspection of connectivity patterns, and the goal of preserving biologically interpretable clusters. Sensitivity checks confirmed that network topology was stable within ±0.05 of the chosen thresholds. A full description of bioinformatic workflows is provided in the Supplementary Materials.

## 7. Conclusions

Our work leverages a modified MEGA-plate to map, in real time, how *Escherichia coli* navigates steep meropenem and gentamicin gradients, uncovering both drug-specific and shared survival tactics. Under meropenem, *E. coli* populations displayed high genetic heterogeneity and pronounced shape changes (filamentation→spheroplast-like forms), consistent with cell-wall stress responses. In contrast, gentamicin elicited a uniform resistance gene set and minimal morphological change, indicating reliance on conserved efflux and enzymatic defenses rather than wholesale remodeling.

Cross-comparison with florfenicol-evolved strains revealed a core resistome of 22 chromosomal loci—among them the global regulators *baeR*, *gadX*, and *marA*—that orchestrate efflux, stress response, and membrane adaptation across antibiotic classes. These master regulators represent high-value targets: impairing them could collapse multiple resistance pathways simultaneously.

By faithfully recapitulating spatial antibiotic gradients, the MEGA-plate bridges the gap between static in vitro assays and the complexity of natural settings, offering a powerful platform for dissecting resistance evolution. Our findings underscore the need for One Health-minded surveillance—tracking shared resistance nodes across human, veterinary, and environmental reservoirs—and point toward combination therapies or adjuvants aimed at these regulatory hubs.

Moving forward, applying MEGA-plate–based approaches to antibiotic cocktails and other pathogens will deepen our understanding of bacterial adaptability. Ultimately, decoding these genetic and phenotypic trajectories is pivotal for designing next-generation interventions that outpace resistance and preserve antimicrobial efficacy.

## Figures and Tables

**Table 1:** Heatmap illustrating the presence (red) or absence (blue) of 64 antibiotic resistance genes (ARGs) in *E. coli* exposed to florfenicol, gentamicin, and meropenem. Each row represents an ARG, and each column corresponds to one of the three antibiotics. The red boxes indicate the detected presence of the corresponding ARG under that antibiotic, while the blue boxes indicate its absence.

	Antibiotic		
ARG	Florfenicol	Gentamicin	Meropenem
*YojI*	1	0	1
*vanG*	0	1	1
*ugd*	1	0	0
*TolC*	1	0	1
*S. flexneri acrA*	0	1	0
*rsmA*	0	1	1
*qacJ*	0	1	1
*PmrF*	1	1	1
*msbA*	1	1	1
*mdtP*	1	1	1
*mdtO*	1	1	1
*mdtN*	1	1	1
*mdtM*	1	1	1
*mdtH*	1	0	1
*mdtG*	1	0	1
*mdtF*	1	1	1
*mdtE*	1	1	1
*mdtC*	1	0	1
*mdtB*	1	0	1
*mdtA*	1	0	1
*marA*	1	1	1
*leuO*	0	1	1
*kdpE*	1	0	1
*K. pneumoniae KpnE*	0	1	1
*K. pneumoniae KpnF*	0	1	1
*H. influenzae PBP3*	0	1	1
*H-NS*	1	1	1
*gadX*	1	1	1
*gadW*	1	0	0
*evgS*	1	1	1
*evgA*	1	1	1
*eptA*	1	1	1
*emrY*	1	1	1
*emrR*	1	1	1
*emrK*	1	1	1
*emrB*	1	1	1
*emrA*	1	0	1
*EC-13*	0	0	1
*E. coli soxS with mutation*	0	0	1
*E. coli soxR with mutation*	0	0	1
*E. coli mdfA*	1	0	1
*E. coli GlpT with mutation*	0	0	1
*E. coli emrE*	1	1	1
*E. coli EF-Tu mutants*	0	1	1
*E. coli ampH*	1	0	0
*E. coli ampC beta-lactamase*	1	0	1
*E. coli AcrAB-TolC with MarR mutations*	0	1	1
*E. coli AcrAB-TolC with AcrR mutation*	0	0	1
*E. coli acrA*	1	0	1
*E. coli UhpT with mutation*	0	1	1
*CRP*	1	1	1
*cpxA*	1	0	1
*baeS*	1	1	0
*baeR*	1	1	1
*bacA*	1	0	1
*ArnT*	0	1	1
*adeF*	0	1	1
*ACT-17*	0	1	0
*AcrS*	1	1	1
*AcrF*	1	0	1
*AcrE*	1	0	1
*acrD*	1	0	1
*acrB*	1	0	1

## Data Availability

The original contributions presented in this study are included in the article/Supplementary Material. The sequence datasets generated and analyzed during the current study are available on NCBI SRA under accession number PRJNA1234206 for gentamycin samples and under accession number PRJNA1234170 for meropenem samples. Please contact the corresponding author for further information.

## Supplementary Materials

The following supporting information can be downloaded at: https://www.mdpi.com/article/10.3390/antibiotics14080841/s1. Figure S1. Schematic representation of the MEGA-plate constructed in AutoCAD (version 2025), illustrating its layered structure and dimensions (54 cm × 40 cm × 25 mm). Table S1. Antibiotic resistance genes (ARGs) identified in the meropenem dataset. A total of 325 ARGs (57 unique genes) were found, conferring resistance to 20 different antibiotics (including penams, penems, carbapenems, fluoroquinolones, and tetracyclines). Table S2. Antibiotic resistance genes (ARGs) identified in the gentamicin samples. Table S3. Significantly enriched gene ontology (GO) terms in the meropenem dataset. Table S4. Significantly enriched gene ontology (GO) terms in the gentamicin dataset. Table S5. Significantly enriched gene ontology (GO) terms identified in the florfenicol-resistant populations analyzed by Kerek et al. [24]. Table S6. Enriched gene ontology (GO) terms for unique ARGs shared among meropenem-, gentamicin-, and florfenicol-resistant populations.
