# Supplementary material for: Deciphering Common Genetic Pathways to Antibiotic Resistance in Escherichia coli Using a MEGA-Plate Evolution System
Source: Antibiotics (Basel). 2025 Aug 20;14(8):841. doi: 10.3390/antibiotics14080841 (PMC12382854; doi:10.3390/antibiotics14080841)
Supplement: Supplementary file 1 [file antibiotics-14-00841-s001.zip › antibiotics-3744415-supplementary.pdf]

# Deciphering Common Genetic Pathways to Antibiotic Resistance in *Escherichia coli* Using a MEGA-Plate Evolution System

**Nami Morales-Durán [1,2], Angel León-Buitimea [1,2], Roberto Álvarez Martínez [3] and José Rubén Morones-Ramírez [1,2,*]**

[1] Facultad de Ciencias Químicas, Universidad Autónoma de Nuevo León (UANL), San Nicolás de los Garza 66455, Mexico; nami.moralesd@uanl.edu.mx (N.M.-D.); angel.deb@uanl.edu.mx (A.L.-B.)

[2] Centro de Investigación en Biotecnología y Nanotecnología, Facultad de Ciencias Químicas, Universidad Autónoma de Nuevo León, Parque de Investigación e Innovación Tecnológica, Apodaca 66628, Mexico

[3] Laboratorio de Biología Cuantitativa y Sistemas Complejos, Unidad de Microbiología Básica y Aplicada, Facultad de Ciencias Naturales, Universidad Autónoma de Querétaro, Santiago de Querétaro 76123, Mexico; roberto.alvarez@uaq.edu.mx

* Correspondence: jose.moronesrmr@uanl.edu.mx; Tel.: +52-818-329-4000 (ext. 3439)

# Supplementary Materials

## Materials and Methods

### *Microbial Strains and Conditions*

*Escherichia coli* ATCC 11229 was used as the model organism for all experiments. Cultures were maintained in Luria–Bertani (LB) broth (Difco Laboratories, USA) and incubated at 37°C with agitation at 150 rpm. Overnight cultures were prepared by inoculating a single colony into LB broth and incubating for 16 hours under the same conditions.

### *Determination of Minimum Inhibitory Concentration (MIC)*

The minimum inhibitory concentrations (MICs) of meropenem and gentamicin were determined using a broth microdilution method, adapted from the Clinical and Laboratory Standards Institute (CLSI) guidelines (Garza-Cervantes et al., 2020; "M100 Ed34 | Performance Standards for Antimicrobial Susceptibility Testing, 34th Edition," n.d.). Stock solutions of meropenem (Laboratorios Química SON'S, Puebla, Mexico) and gentamicin (AMSA Laboratory, Mexico) were prepared according to the manufacturers' instructions.

From these solutions, the necessary volume was added to achieve concentrations of 1 µg/ml of meropenem and 32 µg/ml of gentamicin, respectively, within a final volume of 200 µL. Serial dilutions were then performed by taking 100 µL from each well and adding 100 µL of culture medium, discarding the last 100 µL. Thus, the concentrations tested ranged from 1 to 0.0625 µg/ml for meropenem and from 16 to 0.5 µg/ml for gentamicin. To inoculate each well of the treated plate with the bacteria, an overnight culture (20-hour

culture incubated at 37°C and 150 rpm) of the strain (*E. coli* ATCC 11229) was used and incubated until an optical density at 600 nm (OD 600) of 0.2 ± 0.02 was reached, adjusting it with fresh medium if necessary to achieve the required cell concentration. A concentration range of 107-108 cells/ml was achieved, supported by plate count observations measured by the serial dilution method determined by Garza-Cervantes et al. (2020). From this, a 1:100 dilution was made with fresh medium in a 15 ml tube, and then 100 µL of this dilution was added to each test well to achieve a final concentration of 10^5 cells/ml, subsequently incubated at 37°C and 150 rpm. After 24 hours of incubation under these conditions, the optical densities (ODs) of the control and treated inocula were measured. The MIC determination was the value at which no significant growth was observed (OD 600 < 0.05). All tests and the control sample were performed in triplicate.

*Design of the 40 x 54 cm Acrylic MEGA-plate*

The MEGA-plate experimental setup was adapted from Baym et al. (2016), with modifications to accommodate equipment constraints and optimize experimental conditions. These modifications were made to facilitate handling and maintain a homogeneous temperature (37°C) in the incubator (orbital incubator model: IS-971/ IL-21A) as well as to maintain sterile conditions and be able to manipulate the plate inside the laminar flow hood (model) to avoid contamination, although it is mentioned that with different dimensions, the evolutionary dynamics change (Baym et al., 2016), and the reduction in size did not affect the diffusive growth of the bacteria or the emergence of resistant mutants.

The size of the acrylic plate was changed to 40 x 50 cm. For the base and the lid, an acrylic sheet of 44 cm x 60 cm x 0.6 cm was used. For the walls of the plate, 2 strips of 40 cm x 5 cm x 0.6 cm and 2 strips of 58 cm x 5 cm x 0.6 cm were required. For the 5 intermediate separations, 4 strips of 40 cm x 2.5 cm x 0.6 cm were used. The separation of the 5 bands has a width of 10 cm. As shown in supplementary figure S1, the plate was glued with dichloromethane and sealed with silicone adhesive to prevent spills.

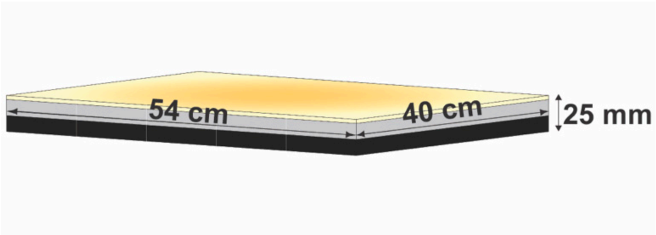


Supplementary Figure S1. Schematic representation of the MEGA-plate constructed in AutoCAD, illustrating its layered structure and dimensions (54 cm × 40 cm × 25 mm). This design ensures a stable environment for establishing controlled antibiotic gradients.

*Sterilization of the MEGA-plate and Culture Media*

Before pouring the media, for each antibiotic, the plate was disinfected with diluted hypochlorite (5-10%) overnight. Six liters of LB medium with a double sterilization cycle were used to reduce the risk of contamination, and these were distributed on the plate in 3 layers (base, intermediate, and surface) with different percentages of bacteriological agar (BD Bioxon). For the base, 2 liters of LB medium with 2% bacteriological agar were used, and kanamycin sulfate (Sigma Aldrich) (30 µg/ml) was added when the medium was tempered to reduce the risk of contamination. To the base and intermediate layers, 4 ml of India ink (Stafford Azor) were added. Subsequently, for the surface layer, the agar

concentration was modified to create a semi-solid medium (0.28%). In this last layer, the antibiotic to be challenged was added at different concentrations according to the minimum inhibitory concentration established for the *E. coli* ATCC 11229 strain, and it was increased to concentrations of 0, 1x, 10x, 100x, and 1000x, respectively.

*Inoculation of E. coli Bacteria*

A 300 μL aliquot of the standardized *E. coli* culture was inoculated along the edge of the lane containing no antibiotic (0× MIC). The inoculation was performed using a sterile pipette tip to create a uniform starting line. The MEGA-plate was incubated at 37°C in an incubator for 9 to 13 days for meropenem and gentamicin, allowing the bacteria to migrate and grow across the antibiotic gradients.

*Selection of Resistant Phenotypes*

Colonies were classified as resistant mutants if they appeared in zones corresponding to antibiotic concentrations higher than the initial MIC (i.e., beyond the visible boundary into the ≥ 1× MIC lane). For meropenem plates, six colonies per lane were selected; for gentamicin, ten per lane. Colonies were chosen at varying distances from the inoculation origin, ensuring ≥ 10 mm separation to minimize sibling selection, and final picks were made using a random number assignment to candidate colonies.

Each isolate was subcultured onto LB agar plates containing the corresponding antibiotic concentration from which it was isolated to confirm resistance (0, 0.125, 1.25, 12.5, and 125 μg/ml) for meropenem and (0, 4, 40, 400, and 4,000 μg/ml) for gentamicin. Additionally, the isolates were grown in liquid LB broth by inoculating them into a 15 ml

Falcon tube with 10 ml of LB medium at the corresponding antibiotic concentration for each lane in triplicate at 37°C and 150 rpm until growth was observed, and the solid culture samples were incubated at 37°C, respectively. All isolates were stored at –80°C in LB broth with 20% glycerol for future analysis.

*DNA Extraction and Sequencing*

Genomic DNA was extracted from overnight cultures of eight meropenem-resistant isolates and ten gentamicin-resistant isolates randomly selected from the resistant colonies described above, using the PureLink Genomic DNA Mini Kit (50 preps, Invitrogen, USA) following the manufacturer's protocol. The extracted DNA samples were then sent to Secoya Labs in the Research and Technological Innovation Park (PIIT) in Nuevo León for nanopore sequencing in accordance with the protocol outlined by (Wang et al., 2021). Secoya Labs specializes in high-quality DNA sequencing and has a state-of-the-art facility that utilizes the latest technology to ensure accurate and efficient results. The quality of the sequenced DNA samples was evaluated based on quality scores and read accuracy. To ensure the accuracy of sequencing data, it is important to carefully evaluate the quality of DNA samples before proceeding with further analysis. This can be accomplished by assessing the quality scores and read accuracy of the samples, as well as by using other methods such as agarose gel electrophoresis or capillary electrophoresis to further evaluate the integrity of the DNA.

*Bioinformatic Analysis*

The complete genomes in the FASTA format were submitted online for evaluation to The Comprehensive Antibiotic Resistance Database CARD, 2024 (https://card.mcmaster.ca/) (McArthur et al., 2013). We used perfect and strict criteria to identify antibiotic resistance genes (ARGs) and uploaded the data separately to the resistance-gene identifier (RGI) platform. Only ARG genes that met the strict and perfect threshold criteria defined by the CARD database and that showed at least 90% sequence identity or coverage were considered. The CARD database combines the Antibiotic Resistance Ontology (ARO) with curated AMR gene (ARG) sequences and various mutations that confer resistance, allowing the identification of ARGs based on annotation and interpretation. This database contains a detailed controlled vocabulary (ARO) with different sequences and mutations of ARGs (Alcock et al., 2023, 2020; Jia et al., 2017). One of the main platforms it uses is RGI, which is CARD's algorithm that enables the bioinformatic prediction of both AMR genotypes and phenotypes obtained from reported genomic data and through the use of bioinformatic models' ARGs (Alcock et al., 2023, 2020; Jia et al., 2017).

To visualize and analyze the interactions between different *E. coli* genome samples, we constructed a heat map and network using the R Core Team program (2024) ("R: The R Project for Statistical Computing," n.d.). The identified resistance genes were then analyzed using the Cytoscape 3.10.2 program (Otasek et al., 2019) and the ClueGo gene ontology enrichment plugin (Bindea et al., 2009). To perform the comparison of the antibiotics gentamicin and meropenem with the antibiotic florfenicol, as well as the bioinformatics analyses, the data were obtained from the article published by Kerek et al. (2024). The Jaccard similarity index (Jaccard, 1901) was used to evaluate the pairwise similarity of gene presence or absence across different lanes, corresponding to varying

concentrations of the gentamicin antibiotic. This index, the ratio of the intersection to the union of genes between any two conditions, quantifies the proportion of shared genes relative to the total number of genes observed across lanes. The Jaccard index was calculated for each pair of lanes representing distinct gentamicin concentrations to determine overlap in the presence of genes. A value of 1 indicated that the same genes were present in both conditions, whereas 0 denoted no shared genes between the two concentrations. This analysis allowed us to compare the genetic profiles across different gentamicin treatments and visualize the degree of similarity or divergence in gene presence patterns. The results are presented as a similarity matrix, a comprehensive tool that identifies potential clusters of genes that respond similarly to antibiotics. The Jaccard index calculation was performed for all possible pairwise combinations of gentamicin concentrations, resulting in a comprehensive similarity matrix. This matrix provides insights into the dose-dependent effects of gentamicin on the gene absence/presence patterns.

Additionally, hierarchical clustering was applied to the similarity matrix to identify group concentrations with similar genetic profiles, potentially revealing threshold effects or nonlinear antibiotic responses. The similarity matrix generated an adjacency matrix with a threshold value of 0.65. Values below the threshold were set to 0, whereas those above the threshold were set to 1. This process yields a binary matrix that facilitates the data representation as a network. In this network, each node corresponded to a lane (indicating a specific concentration of gentamicin), and the connection between nodes indicated a similarity in gene presence profiles that exceeded the threshold. This network structure allows for a quantitative comparison of genetic similarity between conditions and

provides a visually engaging representation, thereby highlighting clusters of lanes with shared gene profiles. This network-based approach enabled the identification of key patterns and relationships within the gene presence data across different gentamicin concentrations. By visualizing the data, we can detect trends, outliers, and potential dose-dependent effects on gene expression. Furthermore, this data representation method can be particularly useful for identifying groups of genes that behave similarly under varying antibiotic concentrations, potentially revealing insights into the shared regulatory mechanisms or functional relationships.

In our analysis of the meropenem dataset, we employed a similar network approach but calculated Pearson correlation coefficients between binary gene presence profiles. Correlation thresholds from 0.35 to 0.65 were tested; lower thresholds produced overly dense graphs, while higher ones fragmented the network. A final threshold of 0.65 was chosen to preserve meaningful sample relationships while minimizing spurious connections. Scores below the threshold were set to 0, and those above the threshold were set to 1.

The analysis involved visualizing, comparing, and interpreting the presence or absence of genes across different antibiotics, such as gentamicin, meropenem, and florfenicol. A heatmap was created using color-coded genes (blue for absence and red for presence) to visually represent gene presence across the antibiotics ("pheatmap function in R," 2021). Subsequently, a more detailed heatmap was generated using the pheatmap library to cluster both the rows (genes) and columns (antibiotics) to observe gene similarity patterns across treatments. A bar plot was then utilized to illustrate the total number of genes present for each antibiotic ("ggplot2 package," n.d.), followed by a stacked bar plot

to depict the proportion of genes present or absent for each antibiotic. Custom color scales were applied to differentiate between presence and absence in the heat maps and bar plots. Lastly, a comprehensive heatmap with row names (gene samples) was generated, clustering rows and columns to elucidate gene presence and absence patterns. This visualization was crucial in providing insights into which antibiotics exhibit similar genetic profiles, observing the relationships between gene presence and antibiotic treatments.

**Supplementary Table S1.** Antibiotic resistance genes (ARGs) identified in the meropenem dataset. A total of 325 ARGs (57 unique genes) were found, conferring resistance to 20 different antibiotics (including penams, penems, carbapenems, fluoroquinolones, and tetracyclines).

**Supplementary Table S2.** Antibiotic resistance genes (ARGs) identified in the gentamicin samples. A total of 378 ARGs (37 unique genes) were found, conferring resistance to 20 different antibiotics.

**Supplementary Table S3.** Significantly enriched gene ontology (GO) terms in the meropenem dataset. Five primary GO categories—positive regulation of gene expression, sulfathiazole transport, response to drug, response to antibiotic, and intracellular signal transduction—were notably enriched, reflecting bacterial adaptive strategies under antimicrobial pressure.

**Supplementary Table S4.** Significantly enriched gene ontology (GO) terms in the gentamicin dataset. As in the meropenem dataset, the same five primary GO categories

were significantly enriched, indicating conserved responses related to gene regulation, transport mechanisms, and intracellular signaling under antibiotic stress.

**Supplementary Table S5.** Significantly enriched gene ontology (GO) terms identified in the florfenicol-resistant populations analyzed by Kerek et al. Seven distinct GO categories were enriched: the methylglyoxal catabolic process to D-lactate via S-lactoyl-glutathione, lipopolysaccharide metabolic process, drug export, response to antibiotic, response to drug, phosphorelay signal transduction system, and sulfathiazole transport.

**Supplementary Table S6.** Enriched gene ontology (GO) terms for unique ARGs shared among meropenem-, gentamicin-, and florfenicol-resistant populations. Six GO terms were significantly enriched: the methylglyoxal catabolic process to D-lactate via S-lactoyl-glutathione, lipopolysaccharide metabolic process, response to antibiotic, response to drug, phosphorelay signal transduction system, and sulfathiazole transport.
